# Surgical technique for the prevention of tracheal necrosis following total pharyngolaryngectomy

**DOI:** 10.3892/mi.2021.18

**Published:** 2021-10-26

**Authors:** Akihisa Tanaka, Hirokazu Uemura, Takashi Masui, Ichiro Ota, Takahiro Kimura, Hiroshi Akioka, Shiori Adachi, Tadashi Kitahara

**Affiliations:** Department of Otolaryngology-Head and Neck Surgery, Nara Medical University, Kashihara, Nara 634-8522, Japan

**Keywords:** tracheal necrosis, total pharyngolaryngectomy, head and neck cancer, inferior thyroid artery, surgical processes

## Abstract

Total pharyngolaryngectomy with cervical esophagectomy (TPLCE) is an invasive procedure with various post-operative complications. Tracheal necrosis (TRN) is a fatal complication of TPLCE. The present study aimed to identify a surgical technique which may be used to prevent TRN. The post-operative complications of 48 patients who underwent TPLCE from January, 2010 to December, 2019 were retrospectively investigated. The incidence of TRN was examined and measures against TRN were reviewed. The results revealed that 3 patients (6%) experienced TRN within 1 week following surgery. In addition, 2 patients required the surgical debridement of the necrotic tissue and tracheoplasty. The other patient underwent conservative treatment. Stomal recurrence developed in 1 patient (2%). On the whole, the present study demonstrates that the incidence of TRN following TPLCE is lower than that observed in previous reports, and only one stomal recurrence was reported. Preserving the blood supply to the trachea is essential for the prevention of TRN. The eight surgical processes used herein effectively preserved the blood supply. Further investigations however, are necessary in order to confirm the present findings and to ensure effective measures are found with which to prevent TRN following TPLCE.

## Introduction

Head and neck cancer, which occurs in the oropharynx, hypopharynx, larynx, or cervical esophagus, is often diagnosed at an advanced stage. Additionally, this type of cancer can lead to tumors which are either synchronous or metachronous. Although total pharyngolaryngectomy with cervical esophagectomy (TPLCE) is a highly invasive procedure, it is the standard surgical and effective treatment for these types of cancer (1-5). In this procedure, tracheal necrosis (TRN) is a common post-operative complication. Previous studies have demonstrated that occurs in 6-20% of patients who undergo TPLCE (6-8). However, there are only a limited number of studies available on techniques that may be used to prevent TRN following TPLCE (6,7). Thus, the present study aimed to identify a technique that may be used to prevent TRN following TPLCE and describes eight surgical processes that may be used for this purpose.

## Patients and methods

### Patient information

From January, 2010 to December, 2019, 48 patients underwent TPLCE at Nara Medical University (Kashihara, Japan). A retrospective analysis of the clinical records was conducted and the incidence of TRN was calculated. Moreover, eight surgical techniques that may be used to prevent TRN were examined. The present retrospective study was approved by the Ethics Committee of Nara Medical University Hospital. Informed consent was obtained from all patients for the publication of the study and any accompanying images.

The patient information and clinical characteristics are presented in Table I. In total, 4 patients were female, and 44 were male. The mean age was 66 years (range, 44-86 years). In addition, 4 patients had oropharyngeal cancer, 34 patients had hypopharyngeal cancer, 5 patients had laryngeal cancer, and 5 patients had cervical esophageal cancer. The grading of the tumors in the present study as per the TNM classification system for cancers is presented in Tables II and III. In all patients, the tissue type was histopathologically confirmed as squamous cell carcinoma (9). The results of p16 immunohistochemical analysis were negative in all patients with oropharyngeal cancer.

A total of 13 patients underwent TPLCE as salvage treatment for recurrent or residual cancer following chemoradiotherapy (CRT) or radiotherapy (RT) alone or recurrent post-surgery. In addition, 7 patients underwent TPLCE following neoadjuvant chemotherapy. Docetaxel, cisplatin and 5-fluorouracil were administered as neoadjuvant chemotherapy. Cisplatin and 5-fluorouracil, cisplatin alone, carboplatin alone, or cetuximab alone were administered as CRT, and the irradiation dose ranged from 60-70 Gy. In total, 19 patients received post-operative RT or CRT due to the histopathological diagnosis, including extra-nodal spread and multiple cervical lymph node metastases. TRN as the necrosis of membranes or cartilage of more than one tracheal ring.

### Surgical treatment

**Figure 1:**
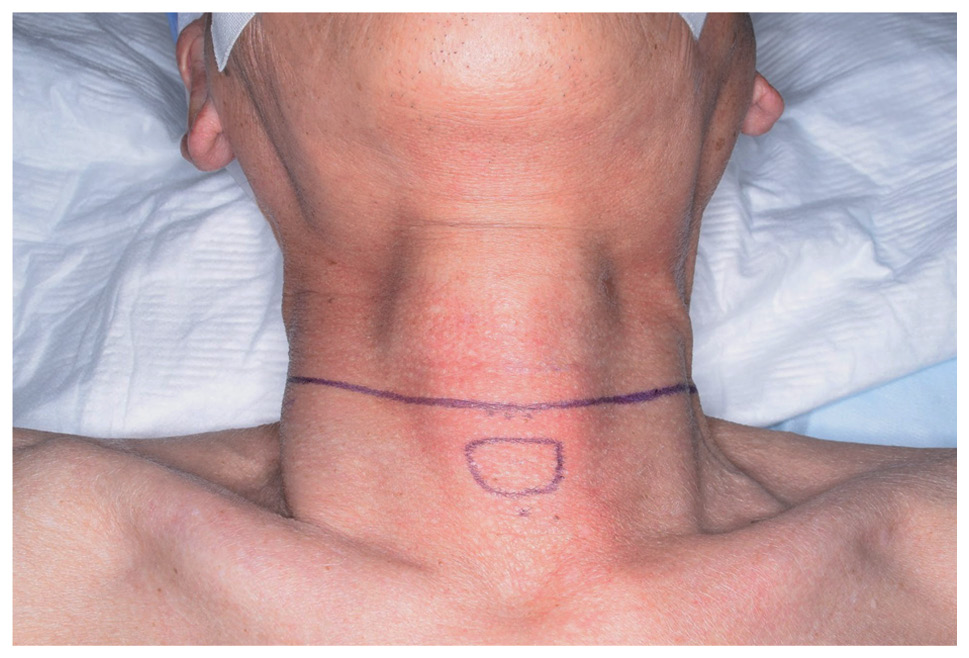
Collar incision.

TPLCE was performed by a head and neck surgeon. All patients underwent reconstruction with a free jejunal transfer, which was performed to compensate for the defect of the cervical digestive tract and was harvested by an abdominal surgeon. Microvascular anastomosis of a free jejunal transfer was performed by a plastic surgeon. Finally, pharyngo-jejunal and jejunal-esophageal anastomoses were performed manually by head and neck surgeons. Although the extent of neck dissection was dependent on the location and size of lymph node metastasis in each patient, all patients underwent at least an ipsilateral neck lymph node dissection and paratracheal node dissection. In this surgery, the cutting lines were set at least 20 mm away from the margin of the tumor to ensure negative surgical margin. Therefore, in all cases, cervical esophagus was included in the resected tissue. In the first process, a collar incision was applied to all patients who underwent head and neck surgery as a cervical approach at Nara Medical University (Fig. 1).

In tumor resections, care is taken so as not to peel the esophageal membrane from the tracheal wall, as much as this is possible, with the exception of cases in which there is tumor invasion to the trachea in hypopharyngeal and laryngeal cancer and in the case of esophageal cancer. At the same time, while peeling this compartment, a scalpel was used instead of an electrocautery. These methods comprised the second process.

**Figure 2:**
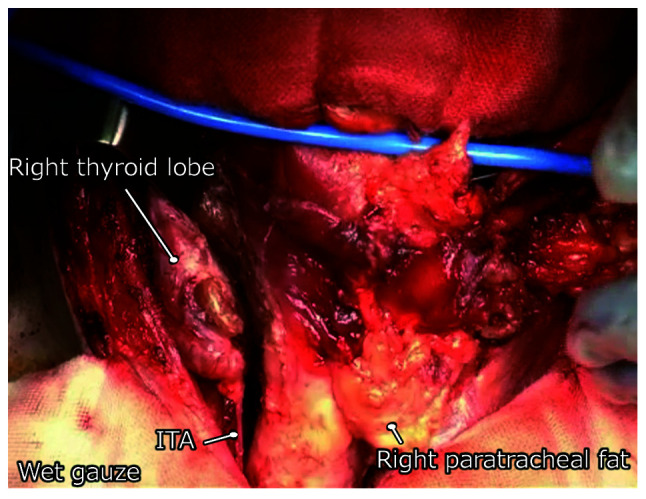
Preservation of ITA. The figure depicts a case with cervical esophageal cancer, which underwent total pharyngolaryngectomy with bilateral neck lymph node dissection and paratracheal node dissection, as well as left thyroid lobectomy. The right lobe of thyroid and right ITA were preserved although the tissue, including the tumor and, left lateral and bilateral paratracheal fat, was resected en bloc (the surgeon is holding the tissue). ITA, inferior thyroid artery.

In the paratracheal operation, care was taken to preserve the branches of the inferior thyroid artery (ITA) as a third process (Fig. 2). When total thyroidectomy or lobectomy is required in some cases, the ITA is cut immediately in front of the thyroid gland. Hemostasis is performed as little as possible, particularly on the surface of the trachea, which indicates the fourth process. Immediately following TPLCE, a small part of the tracheal edge was cut to examine the blood supply to the trachea.

**Figure 3:**
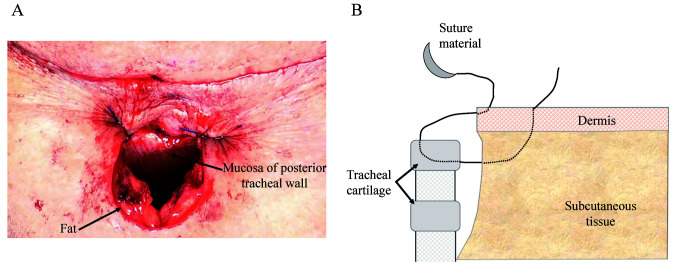
Method for the formation of the tracheal stoma. (A) Design to form the tracheal stoma. (B) Half-buried vertical mattress suture.

When forming the tracheal stoma, the subcutaneous incision after denudation was designed, as shown in Fig. 3A during the fifth process. The half-buried vertical mattress suture with non-absorbable thread was applied to form the tracheal stoma, and the sutures were performed at equal distances, which comprises the sixth process. First, the half-buried vertical mattress suture starts from the epidermal side to the tracheal side. The suture material crosses the wound and tracheal cartilage, similar to a regular vertical mattress suture. Second, the wound on the tracheal side did not cross when returning. Finally, the subcutaneous edge on the epidermal side was crossed, and a knot was made (Fig. 3B).

Surgical loupes magnification was used to view the images more clearly and closely. In addition, the already operated fields were covered with wet gauze to avoid drying. These are routinely performed to preserve the small vessels and are defined as the seventh and eighth processes.

## Results

**Figure 4:**
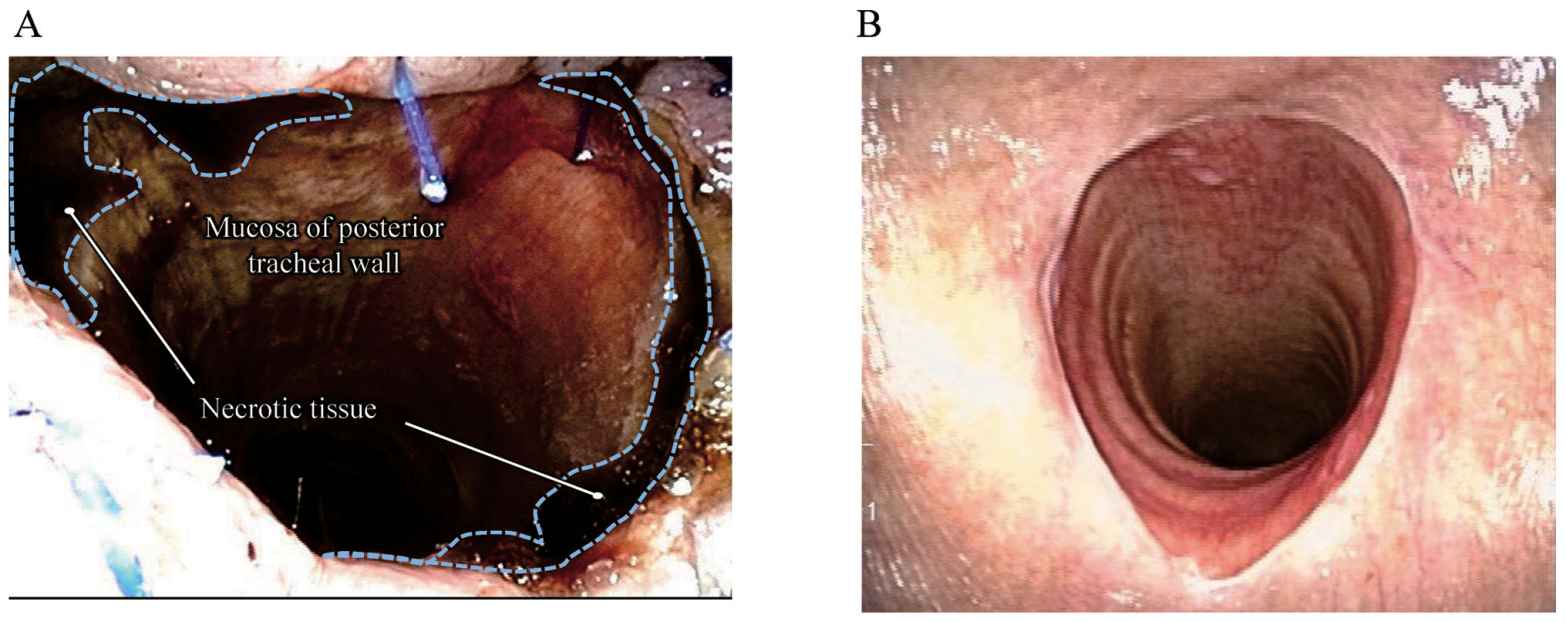
Clinical findings of the tracheal stoma. (A) Tracheal necrosis surrounded by dotted lines. (B) Tracheal stoma at 1 month posts-surgery.

TRN developed in 3 patients (6%) within 1 week following surgery. Additionally, all patients with TRN exhibited necrosis within one tracheal ring (Fig. 4A). The clinical characteristics of the 3 patients with TRN are presented in Table IV. Patient 1 underwent surgical debridement and tracheoplasty. As patient 2 experienced necrosis of the free jejunum simultaneously, tracheoplasty and pharyngostomy were performed using a pectoral major musculocutaneous flap. Patient 3 received only conservative treatments, such as an ointment.

TRN developed in patients 1 and 2 before they received post-operative CRT. TRN did not develop in any of the other patients who received post-operative RT or CRT. Recurrence around the tracheal stoma developed in only one out of the 48 patients (2%).

### Consideration of the patients with TRN

**Figure 5:**
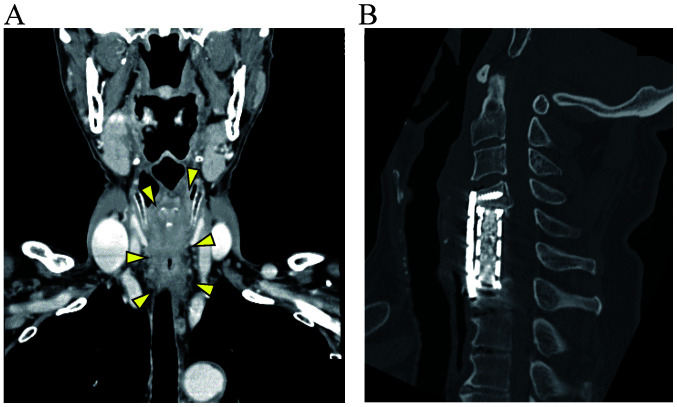
Results of imaging analysis. (A) CT scan illustrating an esophageal tumor in patient 1. The tumor is indicated by arrowheads. (B) CT scan illustrating the plate and screws in patient 2. CT, computed tomography.

The reasons for the development of TRN in the 3 patients in the present study were as follows:

Patient 1 had a cervical esophageal carcinoma close to the tracheal side (Fig. 5A). As the tumor had to be resected up to the closest to the tracheal wall, a number of ITA branches may have become damaged during this procedure, which indicates that the second and third processes were not fulfilled.

Patient 2 underwent anterior cervical discectomy and fusion, and had a plate and screws (Fig. 5B). These materials were close to the tumor that they needed to be removed at the same time. Therefore, the duration of the surgery was lengthy, and the blood supply and tissue may have been disrupted by dryness. Moreover, it was difficult to peel the laryngopharynx and cervical esophagus from the surrounding tissue due to the post-operative use of cicatrix due to anterior cervical discectomy and fusion. In this procedure, several branches of the ITA may have become damaged. A few days after the surgery, the free jejunum was necrotized. Although this patient was salvaged by tracheoplasty and pharyngostomy with a pectoral major musculocutaneous flap, some processes could not be performed, particularly the second and third, in the first surgery.

Patient 3 experienced TRN in only a small portion of one tracheal ring. The preservation of the blood supply to the trachea and the tissue around the tracheal stoma may be insufficient, although care was taken with these eight processes. Fortunately, the TRN improved only by application of an ointment.

## Discussion

*TRN from an anatomical point of view.* TRN is one of the most commonly observed complications following TPLCE and may lead to mortality. Nevertheless, there have been few reports and detailed considerations from an anatomical point of view (6,7). Moreover, a surgical strategy with which to prevent TRN has not yet been established, at least to the best of our knowledge.

The main blood supply to the trachea occurs via the tracheoesophageal branches of the ITA and bronchial artery and the innominate-subclavian system. Although there are some variations in the branching pattern, the ITA is a particularly important conduit for the cervical trachea. The branches of these arteries enter the lateral walls of the trachea. They generally construct lateral longitudinal anastomoses and run transversely around the tracheal circumference to merge branches from the opposite side. The membranous wall receives blood supply from the posterior branches of the longitudinal anastomosis and branches of the esophageal artery (10-13).

*Eight processes for the prevention of TRN*. The preservation of the blood supply to the trachea is essential for the prevention of TRN. The present study described eight processes for the prevention of TRN.

In the first process, a collar incision helps to preserve the supply of blood to the epithelium and maintain postoperative neck flexibility (14). Adding a vertical incision, such as a T-shaped incision, separates the epithelium into several segments, cuts off more blood supply to the epithelium, and does not match the neck striae.

The ITA mainly supplies blood to the cervical trachea, and the preservation of the branches of this artery is crucial (13). The second, third and fourth processes focus on this procedure. Some branches of this artery may be cut by peeling the esophagus from the trachea. Additionally, a scalpel is recommended for peeling, as the heat of the electrocautery may damage these branches. Researchers strive to preserve the small vessels on the surface of the trachea, including longitudinal anastomosis, by preventing unnecessary bleeding and excessive devascularization as much as possible.

Some fats were not removed by setting the Y-shaped subcutaneous incision in Fig. 3A to form the tracheal stoma. These fats receive blood supply from the epidermis, and tissues with blood supply can be applied to the tracheal cartilage. Moreover, the dead space around the tracheal cartilage can be decreased by these fats, which helps preserve local complications such as infection.

The half-buried vertical mattress suture is often used as a surgical technique and it sutures the two sides more closely (15). The suture should be performed at equal distances to ensure equal tension to the stoma. Scar formation and granulation may be prevented by suturing both sides closely (Fig. 4B).

In thyroid surgery, a medical binocular magnifying glass reduces post-operative complications, such as hypoparathyroidism and recurrent nerve paralysis, and is a safe and effective technique (16-18). Considering this evidence, medical binocular magnifying glass would allow for a more detailed viewing, preventing unnecessary vascular damage due to dissection and coagulation by surgical devices.

The tissue is sensitive to desiccation, which may disrupt the blood supply. Dryness has already been shown to delay wound healing (19,20). The dissected site is covered with wet gauze to prevent tissue damage and preserve small vessels whenever the site is not operated on. Intraoperatively, these gauzes were kept moist by sprinkling water at appropriate time intervals.

### Consideration for this study

In the present study, no patients experienced TRN following RT or CRT, and TRN developed immediately after surgery. It was thus considered that post-operative TRN mainly derived from the surgical procedure and these eight processes were played an important role in preserving the blood supply. These eight processes can be applied by any surgeons, and there are few recurrences around the tracheal stoma. In order to prevent TRN, the preservation of the branches of the ITA is essential. Although, in fact, there are some cases with unavoidable factors that may affect the development of TRN, such as thyroidectomy and paratracheal node dissection, the necessity of tracheal resection at a low level and the difficulty in surgery due to pre-operative tracheostomy, care is taken to preserve the blood supply to the trachea with the current surgical technique. The current surgical technique is effective at reducing the risk of developing TRN in cases with these factors as well.

Due to the retrospective nature of the present study, selection bias could not be avoided. Another limitation of the study is the severity of the disease. In other hospitals, surgeons may resect more aggressively owing to the advancement of the disease.

In conclusion, to date, only a limited number of studies have investigated TRN following TPLCE (6-8). The present study described the incidence of TRN at Nara Medical University and the surgical technique used. Knowledge regarding detailed anatomical structures and intraoperative exertion will lead to a decrease in the incidence of TRN. The authors hope that the surgical processes described herein may help numerous patients. Improved surgical techniques for the prevention of fatal complications need to be further investigated.

## Figures and Tables

**Table I:** Background information of the patients in the present study.

Parameter	No. of patients (n=48)
Sex	
Male	44
Female	4
Mean age (range), years	66 (44-86)
Site of tumor	
Oropharynx	4
Hypopharynx	34
Larynx	5
Cervical esophagus	5
Treatment	
Only surgery	14
Surgery with POCRT	14
or PORT	
Salvage surgery after	13
CRT, RT or surgery	
NAC and surgery	2
NAC and surgery	
with POCRT or PORT	5

POCRT, post-operative chemoradiotherapy; PORT, post-operative radiotherapy; NAC, neoadjuvant chemotherapy; CRT, chemoradiotherapy; RT, radiotherapy.

**Table II:** TNM classification of patients with oropharyngeal, hypopharyngeal and laryngeal cancer (n=43).

	T stage					
T stage	N0	N1	N2b	N2c	N3b	Total no. of patients
T2	6 (3^a^)	1	2 (1^a^)	2 (1^a^)	0	11
T3	3 (2^a^)	1 (1^a^)	5	2	1	12
T4a	4 (1^a^)	2	2	9 (1^a^)	2	19
T4b	1 (1^a^)	0	0	0	0	1
Total	14	4	9	13	3	43

The patients were classified according to the UICC TNM classification 8th edition (9).

^a^Indicates the number of patients with recurrent or residual cancer following chemoradiotherapy or radiotherapy alone, or recurrent following surgery.

**Table III:** TNM classification of patients with cervical esophageal cancer (n=5).

	N stage			
T stage	N0	N1	N2	Total no. of patients
T2	0	1	1	2
T3	2 (2^a^)	0	0	2
T4	0	1	0	1
Total	2	2	1	5

The patients were classified according to the UICC TNM classification 8th edition (9).

^a^Indicates the number of patients with recurrent or residual cancer following chemoradiotherapy or radiotherapy alone, or recurrent following surgery.

**Table IV:** Patients with TRN following TPLCE.

Patient	Age, years	Sex	Comorbidity	Site	TNM	Preoperative treatment	Neck dissection
1	63	Male	None	Ce	T2N2M0	NAC	Bilateral neck and bilateral paratracheal lymph node
2	63	Male	None	HP	T4aN2cM0	None	Bilateral neck and bilateral paratracheal lymph node
3	69	Male	DM, HT	HP	T3N3bM0	None	Right lateral neck, left lateral and posterior neck, and bilateral paratracheal lymph node

The patients were classified according to the UICC TNM classification 8th edition (9). Ce, cervical esophagus; NAC, neoadjuvant chemotherapy; HP, hypopharynx; DM, diabetes mellitus; HT, hypertension.

## Data Availability

The datasets used and/or analyzed during the current study are available from the corresponding author on reasonable request.
